# Measuring performance in cancer services during COVID-19 and beyond: a novel case throughput metric using NHS Cancer Waiting Times data

**DOI:** 10.1093/pubmed/fdag041

**Published:** 2026-06-12

**Authors:** Cheuk Him Michael Lam, Thomas Mason, Bruce Hollingsworth

**Affiliations:** Division of Health Research, Faculty of Health and Medicine, Lancaster University, Lancaster, LA1 4YW, UK; Division of Health Research, Faculty of Health and Medicine, Lancaster University, Lancaster, LA1 4YW, UK; Division of Health Research, Faculty of Health and Medicine, Lancaster University, Lancaster, LA1 4YW, UK

**Keywords:** cancer, COVID-19, health services

## Abstract

**Background:**

Cancer Waiting Times (CWT) targets are a central instrument of cancer policy in England, yet conventional performance indicators often obscure changes in service throughput. The COVID-19 pandemic exposed limitations in existing monitoring approaches, particularly for assessing disruption and recovery across cancer pathways.

**Methods:**

A novel case throughput metric (CTM) was developed by extracting National Health Service (NHS) England CWT data spanning 2017–23, covering 334 trusts and an average of 300 000 patients annually. The metric defines service throughput as the ratio of patient volumes meeting waiting time standards during defined periods of investigation normalized to a pre-pandemic baseline, enabling meaningful comparison across trusts while accounting for variation in trust sizes and pre-existing differences in service provision.

**Results:**

The metric revealed substantial heterogeneity in both disruption and recovery across cancer pathways, with diagnostic and referral routes experiencing greater and more persistent throughput loss than treatment pathways. Recovery trajectories also varied markedly between regions, with exploratory analyses suggesting associations with sociodemographic factors.

**Conclusion:**

The CTM provides a policy-relevant complement to existing CWT indicators by making changes in service throughput explicit. It offers a practical tool for monitoring cancer system resilience and informing recovery planning during and beyond major system shocks.

## Introduction

Cancer Waiting Times (CWT) framework introduced by the National Health Service (NHS) England ensures timely access to cancer diagnosis and treatment.^1^ It spans the whole cancer care pathway from referral through to treatment, comprising of nine targets including 2-week targets for timely access to initial consultation, 1-month targets for initiation of treatment following a decision to treat, as well as 2-month targets for the overall time from referral or screening to first treatment.^2^ Performance against these standards is typically reported using total activity volumes or the percentage of patients meeting waiting time targets. Total volumes are heavily influenced by trust size and local population, limiting fair comparison between providers. Percentage-based metrics allow comparison but can mask substantial changes in service throughput, particularly during COVID-19 when referrals and treatments fell simultaneously. For example, Northumbria Healthcare NHS Foundation Trust reported 100% compliance with the 2-week wait breast symptomatic target during the first COVID-19 wave, yet only four patients were seen compared with a pre-pandemic average of around 130 per month. The aim of this study is therefore to develop and apply a metric to quantify disruption and recovery in cancer services in a way that informs policy interpretation, performance management, and recovery planning.

## Methods

### Case throughput metric

The analysis used publicly available CWT data from NHS England, a nationally mandated dataset routinely submitted by all providers, from which monthly trust-level case volumes across the nine targets were extracted for all 334 NHS trusts and independent providers spanning 2017–23, averaging around 300 000 cases annually. A novel Case Throughput Metric (CTM) was then developed to quantify changes in cancer service throughput. For each trust *r* and CWT target *i, CTM_r,i_* was calculated according to Equation 1:


(1) $${\begin{eqnarray*} {CTM}_{r,i}=&\left[\left(\sum_{t=m}^{m+k}\frac{V_{r,i}}{\left|t=m:m+k\right|}\right)\Big/ \left(\sum_{t=n-3}^{n-1}\frac{V_{r,i}}{\left|t=n-3:n-1\right|}\right)\right] \end{eqnarray*}}$$

where:

- *V_r,i_* is the patient volume in trust *r* meeting target *i* within the specified timeframe;
- *m* is the start of the selected window of interest;
- *k* is the duration of the selected window of interest expressed in terms of years;
- *n* is the onset year of the pandemic (i.e. 2020);

This formulation normalizes the volume of patients treated within standard to a stable 3-year pre-pandemic baseline (2017–9), where year-to-year variation was generally within ±3%. The metric essentially compares how many patients were treated within the target timeframe during the pandemic to how many would normally have been treated. A value of 0.5, for instance, indicates that services were operating at half their usual level.

### Analytical approach

CTM was calculated at trust level and aggregated to regional and national levels using volume-weighted averages. The window of interest was defined flexibly to support different analytical purposes, including assessing peak disruption based on the worst pandemic month, and evaluating recovery using aggregated data from 2022 to 2023. Results were summarized descriptively to characterize variation across cancer pathways and regions. Exploratory bivariate analyses were undertaken to examine associations of CTMs with socioeconomic deprivation, ethnicity, and age, identified by literature as key determinants of cancer inequalities.^3^ Deprivation was measured with the Index of Multiple Deprivation (IMD).^4^ Ethnicity (proportion non-White) and age (proportion aged 65+) were extracted from the Office for National Statistics.^5^ Note that these analyses were intended to visualize broad, policy-relevant patterns rather than to establish causal relationships.

## Results

**Figure 1:**
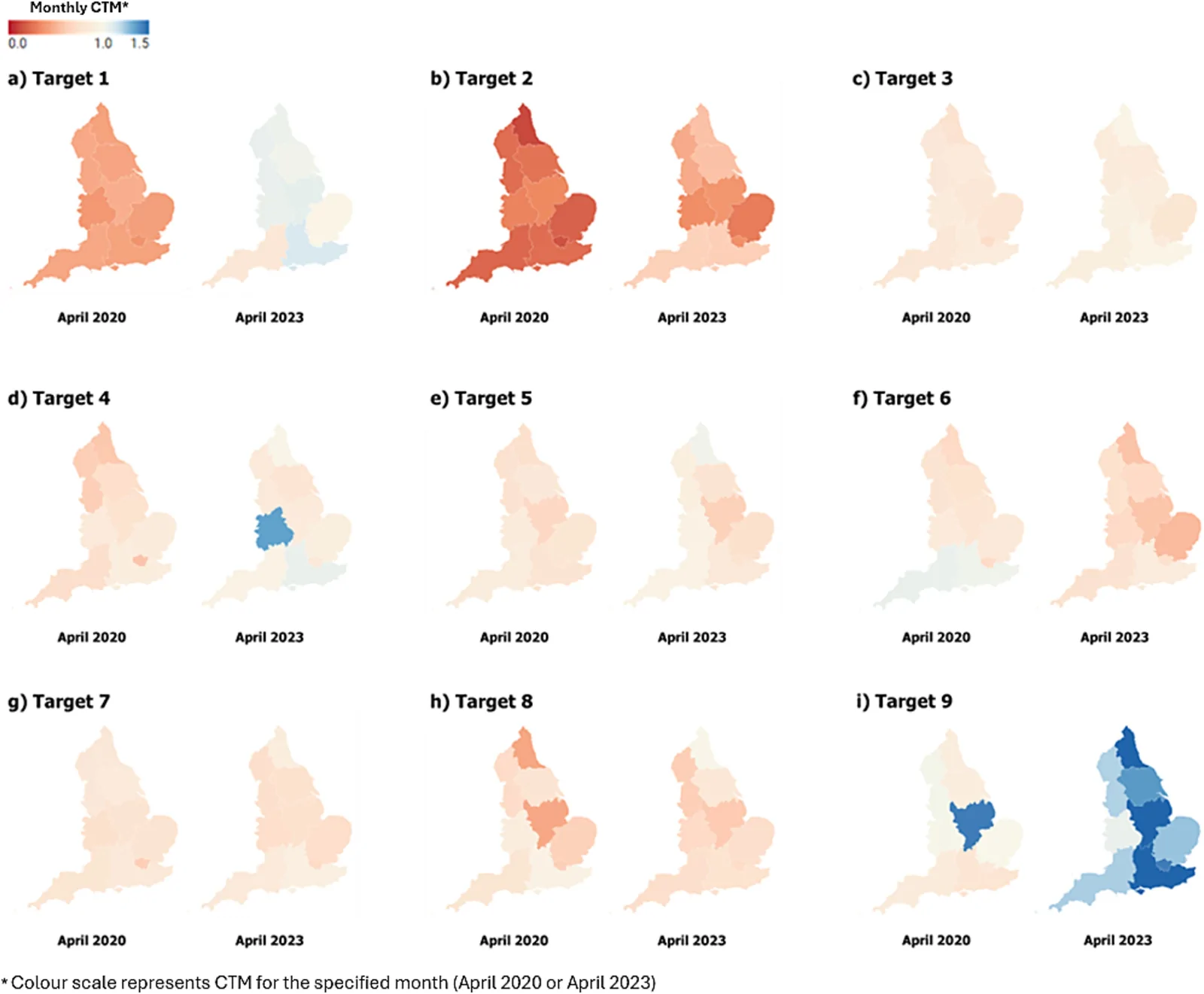
Choropleth maps visualizing monthly CTMs across England for each CWT target in April 2020 and 2023 to showcase recovery progress since the peak of COVID-19, with blue indicating throughput that outperforms the pre-pandemic baseline and red for underperformances, note targets 1–9 correspond to NHS CWT standards: Target 1, 2-week wait from general practitioner (GP) urgent referral to first consultant appointment; target 2, 2-week wait for breast symptomatic patients from GP urgent referral to first consultant appointment; target 3, 1-month wait from decision to treat to first treatment; target 4, 1 month wait from decision to treat to subsequent anti-cancer drug treatment; target 5, 1-month wait from decision to treat to subsequent radiotherapy; target 6, 1-month wait from decision to treat to subsequent surgery; target 7, 2-month wait from GP urgent referral to first treatment; target 8, 2-month wait from national screening referral to first treatment; target 9, 2-month wait from consultant upgrade to first treatment.

Table 1 summarizes the descriptive statistics and regression results for the CTM metrics and the associated sociodemographic characteristics, deviations across regions are further visualized using choropleth maps in Fig. 1. It can be shown that diagnostic and referral targets experienced the largest reductions in throughput during the worst pandemic months, with screening-related pathways showing the most severe disruption. In contrast, treatment-related targets retained relatively higher throughput. Recovery patterns also differed markedly, with several treatment pathways returning to or exceeding pre-pandemic baselines, while referral and screening routes remained below. As for the bivariate analyses, although they were exploratory and based on coarse regional-level data across the nine geographic regions of England, consistent directional patterns were observed across several pathways, highlighting their potential relevance for policy monitoring. Regions characterized by higher levels of socioeconomic deprivation and greater ethnic diversity tended to be more adversely affected and exhibited slower recovery, potentially reflecting lower access to alternative diagnostic routes in deprived populations or cultural barriers in help-seeking behavior. In contrast, regions with older population profiles generally demonstrated comparatively stronger recovery across several pathways, which may reflect prioritization of patients with higher clinical risk within cancer treatment pathways.

**Table 1:** Descriptive statistics and regression results.

*(a) Descriptive statistics of CTM metrics for CWT targets across England’s nine regions (mean ± standard deviation), the standard deviation here indicates considerable regional heterogeneity*									
CWT target	CTM (worst pandemic month)			Occurrence of worst drop			CTM (recovery from 2022 to 2023)		
**Target 1**	0.42 ± 0.04			Apr 2020			1.14 ± 0.14		
**Target 2**	0.23 ± 0.07			Apr 2020			0.54 ± 0.14		
**Target 3**	0.65 ± 0.04			May 2020			1.03 ± 0.08		
**Target 4**	0.73 ± 0.12			Apr 2020			1.07 ± 0.20		
**Target 5**	0.79 ± 0.07			Apr 2020			0.99 ± 0.14		
**Target 6**	0.82 ± 0.09			May 2020			0.84 ± 0.14		
**Target 7**	0.57 ± 0.07			May 2020			0.87 ± 0.10		
**Target 8**	0.05 ± 0.03			Jul 2020			0.82 ± 0.13		
**Target 9**	0.87 ± 0.14			May 2020			1.56 ± 0.33		
*(b) Regression results – Spearman’s correlation coefficients of regional sociodemographic indicators (weighted mean IMD, proportion non-White, and proportion age 65+) and CTM metrics for each of the CWT targets*									
*CTM (worst pandemic month)*									
*CWT target*	*1*	*2*	*3*	*4*	*5*	*6*	*7*	*8*	*9*
**IMD**	−0.27	−0.48	−0.33	−0.63	0.18	−0.51	−0.29	−0.60	0.00
**Proportion non-White**	−0.55	−0.08	−0.27	−0.05	0.25	0.00	−0.72	−0.45	0.13
**Proportion 65+**	0.48	0.18	0.23	0.12	−0.21	−0.17	0.62	0.31	−0.08
*CTM (recovery from 2022 to 2023)*									
*CWT target*	*1*	*2*	*3*	*4*	*5*	*6*	*7*	*8*	*9*
**IMD**	0.07	−0.11	−0.32	−0.32	0.38	−0.29	−0.42	−0.29	−0.45
**Proportion non-White**	0.33	−0.45	−0.18	−0.38	−0.27	0.28	−0.77	−0.24	−0.02
**Proportion 65+**	−0.35	0.39	0.21	0.38	0.18	−0.46	0.70	0.24	0.03

In panel (a), Targets 1–9 correspond to NHS CWT standards: Target 1, 2-week wait from general practitioner (GP) urgent referral to first consultant appointment; Target 2, 2-week wait for breast symptomatic patients from GP urgent referral to first consultant appointment; Target 3, 1-month wait from decision to treat to first treatment; Target 4, 1-month wait from decision to treat to subsequent anti-cancer drug treatment; Target 5, 1-month wait from decision to treat to subsequent radiotherapy; Target 6, 1-month wait from decision to treat to subsequent surgery; Target 7, 2-month wait from GP urgent referral to first treatment; Target 8, 2-month wait from national screening referral to first treatment; Target 9, 2-month wait from consultant upgrade to first treatment.

In panel (b), higher values of the sociodemographic indicators reflect generally less advantageous population profiles (i.e. greater deprivation, larger ethnic minority populations, and older age structures). Negative associations (blue) indicate better throughput in more favourable sociodemographic contexts, whereas positive associations (red) indicate better throughput in less favourable contexts.

## Discussion

### Main finding of this study

This study introduces CTM as a complementary indicator to conventional CWT targets, demonstrating its ability to reveal changes in service throughput that are not captured by percentage-based compliance measures.

### What is already known on this topic

Cancer care performance is assessed using a range of indicators, including waiting-time standards (i.e. CWT), waiting list size, and counts of completed pathways or activity volumes.^6^ However, they are typically reported in isolation and do not directly reflect changes in underlying service throughput relative to expected levels.

### What this study adds

CTM quantifies relative throughput by comparing activity to pre-pandemic baselines. Take Northumbria Hospital as an example, 100% compliance can coexist with substantial reductions in activity (CTM as low as 0.03); such discrepancies are likely masked in standard reporting. This is particularly relevant during periods of system stress, including pandemic disruption, industrial actions, or budget constraints. CTM therefore supports more targeted policy responses and can be implemented using existing datasets with minimal additional burden.

### Limitations of this study

With CTM, it may be difficult to disentangle changes in demand and capacity, particularly when they occur simultaneously. Observed changes may therefore reflect both demand- and supply-side effects, a limitation inherent to most existing metrics. As with any other measure, CTM warrants careful interpretation, requiring sufficient data literacy and complementary indicators to reduce the risk of misjudgment.

## Conclusion

CTM provides a practical tool for monitoring disruption and recovery during major system shocks. Incorporating throughput-based metrics alongside conventional targets could strengthen performance interpretation, support recovery planning, and improve preparedness for future pressures on cancer services.

## Data Availability

The data underlying this article are publicly available from NHS England Cancer Waiting Times statistics at: https://www.england.nhs.uk/statistics/statistical-workareas/cancer-waiting-times/.

## Notes

**Cheuk Him Michael Lam**, Doctoral Researcher in Health Economics and Policy

**Thomas Mason**, Senior Lecturer in Health Economics

**Bruce Hollingsworth**, Professor in Health Economics
